# Early Maladaptives Schemas among call center staff in the Rabat Sale Kenitra region, Morocco

**DOI:** 10.1192/j.eurpsy.2022.1814

**Published:** 2022-09-01

**Authors:** E. Drissi, S. Boulbaroud, H. Hami, A.O.T. Ahami, F.Z. Azzaoui

**Affiliations:** 1 UIT, Biology, Kenitra, Morocco; 2 Sultan Moulay Sliman University, Bioogy, benimellal, Morocco

**Keywords:** Morocco, Early maladaptives schemas, call centers, employees

## Abstract

**Introduction:**

It is important to know the prevalence of the Early Maladaptives Schemas (EMS) in such population.

**Objectives:**

The study of Early Maladaptives Schemas among call center staff in the Rabat Sale Kenitra region and possible socio-economics factors that may influence them.

**Methods:**

The study involved 121 call center’s employees in the Rabat Sale Kenitra region. They responded to an informative questionnaire and to the SPI 26, with 26 items, including 13 early maladaptives schemas.

**Results:**

121 subjects were interviewed, 48.78% (n=59) men and 51.24% (n=62) women, a minimum age of 22 years, a maximum age of 60 years and an average of 31.74 7.93. Through the examination of the EMS’s results in adulthood, we note a decreasing ranking of active shemas according to the rate of participants: the EMS Unrelenting standards is active in 80.02% of our sample, the EMS Mistrust in 61,2%, the EMS Insufficient self-control in 47.9%, the EMS Abandonment in 47.1%, the EMS Insufficient self-control in 41.3%, the EMS Emotional inhibition in 38.8%, the EMS Vulnerability to harm or illness in 33.1%, the EMS Dependence in 31.4%, the EMS Selfsacrifice in 27,3%, the EMS Social Isolation in 19%, the EMS Emotional deprivation in 10.7%, the EMS failure in 8.3% and the EMS Enmeshment in 7,4%.

**Conclusions:**

By comparing the rates of EMS in childhood and adulthood, it emerges that only the EMS Abandonment, Dependence and Insufficient self-control showed a disinensification, increasing successively from adulthood to childhood as follow :from 69.4% to 47.1% , from 52,9% to 31.4% and from 59.5% to 47.9%.

**Disclosure:**

No significant relationships.

